# Second Examination of Mrs. Cumming. On the 9th Day—January 16th

**Published:** 1852-04-01

**Authors:** 


### SECOND EXAMINATION OF MRS. CUMMING.

On the 9th Day—January 16th.

Mrs. Cumming.—There are more of you than this room will hold. I thought there were only twelve gentlemen coming.

The Commissioner.—There are not many more. I am afraid they are all entitled to be here.

Mrs. Moore.—Gentlemen, I was requested by Dr. Caldwell to say that Mrs. Cumming was very much fatigued by the last visit.

Foreman of the Jury.—We wish you to retire (referring to Mrs. Moore and another person in the room.)

Mrs. Moore.—I understood that I was to remain with Mrs. Cumming.

Foreman of the Jury.—No, you must retire, if you please.

The Commissioner (to Mrs. Cumming).—You were in Wales a year ago? A. Yes, I was.—Q. And saw all your property there? A. Yes.

A Juryman (to Mrs. Moore and the other female).—You will leave the room, if you please, both of you.

Mrs. Cumming.—But I require that lady (referring to Mrs. Moore).

Foreman of the Jury.—We cannot allow it.

The Commissioner (to Mrs. Cumming.)—Who do you wish to have here?

Mrs. Cumming.—One of my attendants, or that lady.

The Commissioner.—Dr. Caldwell is here.

Mrs. Cumming.—Dr. Caldwell is a medical man.

The Commissioner.—He is your medical man; he will be here close at my elbow; and if he thinks I am doing wrong, he will tell me so. I have told Dr. Caldwell to be close to my elbow with that view.

Mrs. Moore.—I may remain here, surely?

A Juryman.—No.

The Commissioner (to Mrs. Cumming).—They may go out, may they not?

Mrs. Cumming.—Sir, that lady is living here.

Mrs. Moore.—I was desired to attend Mrs. Cumming, and not to leave her. Surely you will let me remain?

Dr. Caldwell (addressing Mrs. Moore and the other female).—You are doing Mrs. Cumming great harm.

The Commissioner (to Mrs. Cumming).—You do not require them to remain, do you?

Mrs. Cumming.—I do.

The Commissioner.—When we were here before, they left the room, and you did not object?

Mrs. Cumming.—No; I was not aware of the inconvenience I should be placed in.

The Commissioner.—I am very sorry that you should have been placed in any inconvenience. I know we were rather long. What was the inconvenience we placed you in, except disagreeableness of seeing us?

Mrs. Cumming.—I did not know that you all were so ugly as that.

The Commissioner.—If there is any gentleman you see here who you would wish to retire, perhaps you will tell me. Is there any gentleman here you would wish to retire?

Mrs. Cumming.—No; but any sensible person would draw a line between having a female in the room and a medical man.

The Commissioner.—Would you wish one of these females to be here?

Mrs. Cumming.—I wish one of them to remain.

The Commissioner.—Which lady would you prefer?

Mrs. Cumming.—That lady (pointing to Mrs. Moore).

The Commissioner.—Just go behind, Mrs. Moore. (Mrs. Moore, who had been previously standing opposite to Mrs. Cumming at the door, took a seat at the window).

The Commissioner (to Mrs. Cumming).—In what way did we annoy you the other day? A. If you wish to know the rights of it, ask my medical man; he is more competent than I am to answer you.

The Commissioner.—I will not press the matter further. A. I think delicacy ought to forbid your doing so.

The Commissioner.—I hope you will not think I pressed you unfairly the other day. A. You have a right to think so. — Q. You went into Wales last year? A. Yes, I did. — Q. You went to see your property? A. I did. — Q. Do you recollect how long you were there? A. I was three or four months in Wales. — Q. You took some time to go. How long were you in getting down there? A. I went by easy stages. Q. In your own carriage, and with your own coachman? A. Yes. — Q. You had Miss Miller, I think, with you? A. Not from London, I had not. — Q. She did not go with you from London? A. No. — Q. You rented a house? A. I had a house to myself there. — Q. Bassaleg was one; and you had one also at Newport? A. I did not go to my own house. Q. But you rented one of Mr. Edwards, was it?

A Juryman.—Evans? A. Evans. — Q. Do you remember what you were to give for it? A. There was no stipulated sum mentioned. — Q. What were you to pay a-week for it? A. No, I said I would give him a compensation when I came away; he went out with me himself, and went to his sister's, who was next door. — Q. Did you not go to your own tenant's at Blackbird's Nest, and meet him there? A. No; whoever told you that told a gross falsity. — Q. He tells us he saw you at Blackbird's Nest? A. So he might, sir; I went several times there. — Q. And he says you went from his house to look at it? A. I might have looked at it. I did not go there as lodgings. — Q. Do you know what you were to give him for it, for the house of Mr. Evans? A. About 60*l.*; but there was no specific sum mentioned. He asked me to come to his house, if I would put up with it such as it was. I told him I was very much obliged to him indeed. I should not have gone at all, only the cholera was raging very much about Newport. — Q. Had not you some difference with the landlady of the house you were in—Mrs. Phillips? A. It was about a piano. — Q. We are told so? A. That is correct, sir. — Q. He said you were to pay thirty-five shillings a-week for the rent of the other house you had when you quitted Mrs. Phillips? A. There was nothing of the kind, but I was to give him compensation, and he said, "Never mind, Mrs. Cumming, you are welcome to it if you can put up with it such as it is." — Q. Do you remember going to the Rev. Dr. Williams, the chancellor of the diocese? A. Yes. — Q. You remained there an evening? A. Yes, and called there frequently. — Q. Do you remember how often you called there? A. No, because I should have thought such a trivial circumstance as that would not have been worth mentioning. — Q. You had a pleasant evening there three or four times? A. Yes, very pleasant. They were very genteel people. — Q. Do you remember saying anything about your daughters? A. There were many people in the room, there might be general conversation. — Q. Do you remember saying anything to Dr. and Mrs. Williams about your daughters? A. It was such an insignificant subject that I did not bear it in mind. — Q. I was in hopes that your daughters were not insignificant? A. To me they are obnoxious—I cannot use a stronger term than that. — Q. We have heard of an arrangement which was entered into in 1846? A. Yes, you were the commissioner then. — Q. I did not interfere about the arrangement? A. I did not say you did. — Q. Why did you break it off? Do you remember? A. Because I had the property in my own hands, and I had no occasion to ask for what I had got. — Q. Did any particular person recommend you to do it? A. No. When I found out what was kept in the back ground from me, it was necessary that I should change my mind. — Q. How did you find out the difference, for I have not seen the papers to ascertain what it really was. When was it that you discovered you were under a mistake? A. When I heard it publicly named to me that my father had left it all to myself, which was the case. — Q. You do not remember when it was that you found out that you were entitled to it absolutely, instead of for life. Do you remember when you first heard that and made that discovery? A. It must have been nearly about the time when I was brought into court, because I could not have much opportunity when I was locked up in a madhouse, put there and kept there by my daughters. — Q. You said that when the agreement was entered into you thought you had only the property belonging to you for life, but you afterwards discovered that you had got it absolutely? A. Exactly so. — Q. Do you know when you found out that you had a greater interest than you thought you had? A. When the brief and papers were looked into. Q. You do not remember how long afterwards? A. Very soon afterwards. — Q. You do not remember where you were at the time? A. I remember that; my memory is not so treacherous as all that. — Q. Why did you not try at that time to come to terms with your daughters, and to make up past grievances? A. Because I had too much of them as it was. — Q. Between parent and child would it not have been desirable? A. No, sir; not all the powers on earth would ever induce me to alter my determination, never. — Q. We can only regret it. You have a right to entertain what opinions you think fit. I took the liberty of asking you the other day what property had been sold. Do you remember what property you sold to the Railway Company? A. I remember all that, but I believe that is a thing private to myself, which no gentleman here can be interested in. — Q. We wanted to see whether what you stated the other day was quite accurate. Can you tell us at all what was sold to the Railway Company? A. I could if I chose, but I do not think that any one has a right to ask the question; I tell you that candidly.

The Commissioner.—You can refuse to tell me. A. I should not like to behave rudely to you as a gentleman. — Q. I should not consider it as rude. Can you tell us what you sold to the Water Works Company? A. I could do all that if I chose.

A Juryman.—I wish you would understand we are here for your benefit, and the more correctly you give us information, the better we shall be satisfied of your capability of doing it.

Mrs. Cumming.—That is a private concern of my own.

The Commissioner.—I am afraid our coming here is private.

Mrs. Cumming.—Oh, it is public enough all through the neighbourhood, and through London, I believe. — Q. You do not think we come as enemies? A. That I cannot tell. — Q. These gentlemen are not they perfectly disinterested?

A Juryman.—Your answers to us will decide our view. We wish every justice done to you.

The Commissioner.—I should be very glad to hear any observation you would like to make to these gentlemen; it would be much more gratifying to me than my putting questions to you. You may naturally feel and think that I am intruding on you.

A Juryman (to Mrs. Cumming).—You would oblige us very much if you would state what your property was sold for? A. No; I would not state that, most decidedly.

A Juryman.—Do you have an account at your banker's? A. Yes.

The Commissioner.—Will you let us look at your banker's book? A. No, sir, I thank you. — Q. Did you have a banker? A. I did.

A Juryman.—What was the name of the banker? A. Do you think they would, without having an order from me, give up anything?

A Juryman.—No; that is not likely. What is the name of your banker? A. Scott.

A Juryman.—Do you recollect a Mr. Haynes in Palace Chambers? A. I do, perfectly well. Q. What is his name; is it Robert? A. No, it is not. — Q. What is his name? A. I always called him Mr. Haynes. — Q. You do not know his Christian name? A. No. — Q. Do you recollect any transactions you have had with him? A. Yes, I do.

The Commissioner.—Do you remember his paying you a considerable sum of money one day in a carriage? A. No, not in a carriage.

A Juryman.—Was any money paid to you in Mr. Haynes's office?

The Commissioner (interposing).—Not Robert Haynes, but Joseph Haynes? A. I know what you mean—his brother.

A Juryman.—Did you ever receive any money in his office? A. Yes, he has paid me money.

The Commissioner.—Was that for the sale of some property? A. I have had money from him several times. — Q. He once gave you a cheque for 20*l.*, and at another time for 24*l.*, but the gentleman is referring to a larger sum?

A Juryman.—Do you remember receiving a larger sum for selling an estate of yours in Wales? A. Yes. — Q. Do you know what amount you received? Do you recollect the sum of money you brought home with you in the carriage? A. Yes. — Q. Will you tell us?

The COMMISSIONER.—In round figures? A. About 300*l.* — Q. Are you sure of that? A. Not for one estate, you know.

A JURYMAN.—Did you receive it at his office, 300*l.*? A. I think I did. — Q. Did you take it to your banker's? A. Not directly.

The COMMISSIONER.—In what shape did you receive it? Do you recollect whether he gave it you in a cheque or in bank-notes. A. Principally in bank-notes.—Q. Not in a cheque for a round sum? A. Upon my word I cannot tell. To tell you the truth, I did not know I should be called to account, because that is a private affair of my own. — Q. I am afraid the questions put to you are private. I asked you the other day, and did not like to press you. It seems rather important we should know, for your sake at all events, what mortgage there is upon these two houses; we understand there is a separate mortgage upon each. Do you know the total? A. Yes, I do. — Q. Cannot you tell us? A. If I chose, I could. — Q. Will you have the kindness to tell these gentlemen? A. No, you must excuse me, if you please, for that I consider my private concern.

A JURYMAN.—Have you the deeds of this house in your possession?

The COMMISSIONER.—Do you keep your title-deeds and plate in this house? A. I should be sorry indeed to do it—to keep them in the house.

A JURYMAN.—Will you allow me to ask, do you know where the title-deeds are? A. I can allow you to do that; but I can refuse to answer that question.

The COMMISSIONER.—These gentlemen are sorry you should be on bad terms with your children. Do you remember that Mr. Ince or some member of your family got some saltcellars once belonging to you? A. No, I do not. — Q. It is said that you entertained the impression that one of them had taken some saltcellars belonging to you? A. I never said so.

A JURYMAN.—A silver basket? A. Yes, sir, a little basket he had.

The COMMISSIONER.—How did he get possession of it; do you know? A. I do not know how he got possession of it. — Q. Did he not send it to you, or did not Mrs. Ince send it to you? A. After it was asked for a great while. — Q. What made you think they took it away from you, or stole it? A. Stole it! I did not make use of that expression. — Q. Had you the impression they took it away from you? A. I do not know whether it was a delusion or no, but Mrs. Ince brought it back to me. — Q. Do you know how they got it? A. Not given by me. — Q. Was it not sold amongst some things that were taken away under the distress put in for Mr. Cumming's debts, or something of that kind? A. It was never sold in a public court, not to my knowledge: and there is his gold watch, I have not got that. — Q. Who do you suppose has that? A. I suppose it is at the pawnbroker's, if Benjamin Hooper has not got it away, for he took it there. — Q. Now, these saltcellars which Mr. Ince redeemed from some pawnbroker's, do you remember those? A. He redeem them?— Q. I was told so? A. Then you were told a gross falsity. — Q. What is your impression of the state of things with regard to those saltcellars? A. I did not think much about them afterwards. — Q. Did he not redeem them for you? A. My saltcellars? — Q. Yes? A. He, never; he had it not in his power to do it. — Q. He had not the money? A. I did not say he had not the money. — Q. Did you give him the money to do it; did you give him the money with which to redeem them. You seem to be under the impression that he did something wrong about them? A. No, I have not said so. — Q. You are under no impression of that kind? A. No. — Q. Do you remember who introduced Mr. Thorne to you as your solicitor? A. I do. — Q. Can you tell us who it was? A. If it is of material consequence I will tell you. — Q. As you are asked, perhaps you will have the goodness to answer it? A. I believe I am not forced to answer every question that you put to me. — Q. Certainly not, but do you mind telling us who introduced Mr. Thorne to you? A. It does not redound much to his respectability when I tell that. — Q. As far as I know, he is a respectable man? A. Oh, sir, he is a very respectable man for those who like to employ him. He is at anybody's serviee for me now. — Q. You seem to doubt whether he is so? A. No, I do not doubt it, I only speak feelingly. — Q. In what respect has he neglected your business? A. I never put it in his power to. — Q. Was he not your solicitor for some time? A. No, only for a few days, or a week. — Q. Not more than that? A. No. — Q. I thought he had been your solicitor for some months? A. He might. I was in very bad health, and I only requested him once to call on Mr. Robert Haynes about some moneys that I wanted. — Q. Do you remember Mary Ann Hickey being there? A. She was a little girl here, I seldom saw her.— Q. Used she to read the newspapers to you? A. She would spell them to me as well as she could, if that may be called reading. — Q. She was very young at that time? A. She has had time for improvement now. — Q. Do you remember holding a knife in your hand and threatening her? A. No, that is an infamous lie. — Q. You never did so? A. Never. — Q. Because she intimated something of that kind? A. There was a great deal intimated; others have intimated it. She was a mere child. — Q. You have employed Messrs. Carlon and Haynes as your solicitors, I think? A. Yes, I have. — Q. Do you know whether you owe them anything or not as solicitors? A. I believe I do. — Q. Do you know how much? A. No, we have not settled our accounts. — Q. Have they sent you in a bill? A. I do not know, I have not seen it, because it is customary when you have a solicitor for another solicitor to send the bill in to him; that is the case for gentlemen, I mean, much more for ladies. — Q. You think Carlon and Haynes have not sent you their bill; do you think they have sent it to anybody else? A. I do not know, I can only answer for them that they are respectable solicitors. — Q. You say Captain Haynes's watch? A. Cumming's, I beg your pardon. — Q. Yes, I beg your pardon; that Captain Cumming's watch is in pawn now? A. I do not know that it is, but it was put in pawn for him by Benjamin Hooper. — Q. And you have not seen it since? A. I have never seen it since. — Q. You remember a draft-will which you gave directions to be made? A. Yes, I do. — Q. Do you remember mentioning anything about the watch there? A. Probably I might, but that is destroyed now. — Q. It was in writing, you know? A. Yes, but it was never signed. — Q. Do you remember what money you gave in that will to Mr. Haynes? A. Various sums I have given. — Q. But to Mr. Robert Haynes himself, you do not remember? A. I do not recollect at this moment, because the will is destroyed, you know. — Q. But you have a copy of it, which I showed you the other day? A. I have not got it. Mr. Thorne might give it you, he took it away meanly and pitifully. I thought he was a gentleman, you know. — Q. He took it away and produced it before me? A. Yes, he did, but he would not tell you how he got it, though. — Q. I think he said you gave it him? A. Not to take it out of the house. If you were to let me look at one of those papers, (referring to some papers in the Commissioner's hands,) would I take the liberty of not returning it to you—no, I would scorn it as a gentlewoman. — Q. You say you destroyed the will? A. Yes. — Q. What will did you destroy? A. The will I made when I was very ill. — Q. And you signed it? A. No.

A Juryman.—You never made a will at all? A. Not besides that. — Q. There is no will in existence now? A. Not to my knowledge. — Q. It must be to your knowledge if you made one? A. Exactly so, so it would.

The Commissioner.—Do you remember whether that silver basket, which we have talked about, was ever put up for sale? A. No, it never was. — Q. Did not your daughter, when you were at Greenwich, write you a letter offering to buy anything for you? A. I do not remember. — Q. About the time the things were seized, and sold in consequence of some debts your husband had incurred, I want to set your daughter right in that respect; do you remember her writing you a letter offering to buy in anything for you? A. I had employed a person. — Q. A person at Greenwich? A. Yes. — Q. To buy in something for you? A. Yes. — Q. Do you remember what it was he bought? A. Nothing very valuable, only what I set a value on. — Q. Family? A. Plate I may call it, plate, you know. — Q. What was it? A. It was of very little value. — Q. But you do not wish to tell me what it was; you told me the other day that the property you sold to the Railway Company altogether amounted to 7000*l.* or 9000*l.*, that is, what you sold to the Railway Company and the Water Works Company, do you remember at all how much you did sell? A. Why, sir, I was not down in Wales then. — Q. When the thing was going forward? A. No. — Q. But can you remember the figures; can you remember what the Railway Company were to give you. There was a house which you would not let them have without the garden, and then they took the house and garden? A. The house and premises. — Q. What were they to give you for that? A. They were to give me 2000*l.* for that. — Q. Were they to give you anything for anything else, the same company. A. Not at that period, it was at another period. — Q. What were they to give you for the other property that was sold afterwards? A. It was nearly 8000*l.* — Q. To whom were you selling? A. To the Railway Company. — Q. I understood you to say so the other day?

A Juryman.—6000*l.* she says.

Mrs. Cumming.—That is what I told you the other day.

The Commissioner.—Then you sold some to the Water-works Company besides? A. Yes, at different periods, you know. — Q. Do you recollect what the Water-works Company were to give you? A. Nearly 1000*l*.

A Juryman.—Then there were Sir Charles Morgan? A. That is lately.

The Commissioner.—I understood you do not approve of that sale to Sir Charles Morgan? A. Who said so? — Q. Do you know what you are to receive from him? A. Yes; perhaps it is received now for what I know. — Q. Do you know what the sum is? A. If you can tell me what the premises were, then I will tell you.

A Juryman.—It was somewhere near Tredegar, among which there was a piece of land which the clergyman of Bassaleg had, Mr. Williams. It was put up for sale once, then bought in, and then you sold it to Sir Charles Morgan? A. That is truth, whoever told you that. — Q. What was he to give for it? A. It was only a little bit of ground round the church.

The Commissioner.—Was that all you sold to Sir Charles Morgan? A. I cannot charge my memory with all that, because there has been a good deal of my property sold before I came into it, to Sir Charles. — Q. But since the year 1846? A. There has been none sold to Sir Charles unless it is without my knowledge. — Q. Since 1846 there has been none sold without your knowledge, I hope? A. No; that was not sold without my knowledge. — Q. What have you sold to Sir Charles Morgan since 1846? A. Nothing since 1846. — Q. I understood you to say so the other day, excepting Sir Charles Morgan, or Morgan and Bailey, a bit of land? A. Yes; but that is a different one from this: it is a very common name in Wales, Morgan is. — Q. Do you think there has been some sold to Morgan and Bailey? A. No; not Morgan and Bailey.

A Juryman.—The Rev. Mr. Williams is not your tenant now? A. He never was my tenant. — Q. He was when you went there in 1849? A. Yes; that is some years ago. — Q. The property has been sold to Sir Charles Morgan? A. Yes; but Mr. Williams keeps it. — Q. But he is not a tenant to you.

The Commissioner.—You do not get the rent? A. No; I could not expect the rent when I had sold it. — Q. What did you sell it to Sir Charles Morgan for? A. It is easily told, because it is a very little bit. — Q. There were from two acres and a half, to two acres and three-quarters? A. Yes. — Q. Can you tell us at all what you did sell to Sir Charles Morgan, or any other gentleman of the name of Morgan? A. Round the church; that was a small spot of land that I sold to Bailey. — Q. The receipts which you have given for your rent—do you draw them out yourself? A. I do. — Q. The whole of them, body and all? A. Yes; but since my eyes have been so bad I only *sign* them. — Q. How long have you given up writing the body of the receipts? A. Not long; perhaps I may do it again when my eyes get better—I have a dreadful cold in my eyes. — Q. The cheques on your banker, do you draw them? A. I do sometimes, according to the state of my health. — Q. Do you remember seeing Mr. Williams in your carriage, and receiving his rent up to 1849, and giving him a receipt? A. Yes, I do; but I gave him a receipt from myself then. — Q. They were all signed by you? A. Yes.

A Juryman.—When was it you gave him the receipt in the carriage? A. When I received the money. — Q. When was that? A. I do not think that is a question I have a right to answer. — Q. Do you ever have your banker's book sent home and made out? A. No; because I call there very often. — Q. When was it last made up? A. Why, very lately. — Q. About what time do you have it made up: at particular periods? A. You know I cannot have access always to the banker's. — Q. But you can send? A. Yes; but we should be very tenacious who we send. — Q. Can you not trust the people about you? A. Yes, I can. — Q. Do you think it was made up at Christmas? A. There is not a great deal in it, because I have drawn it myself. — Q. How often do you generally get it made up? A. Not at any particular period. — Q. You have some grandchildren? A. I believe so. — Q. They have given you no cause of displeasure? A. I never put it in their power. I have quite sufficient to occupy my mind by my own children, their parents. — Q. I understand you to say, you saw Mrs. Ince sometimes out of your window? A. To be sure, I could not do otherwise when she came there and pestered me from morning till night. — Q. Was she alone when you saw her? A. I cannot tell that. — Q. You would see if other people were with her? A. Not if they stand behind. — Q. How often have you seen Mrs. Ince out of these windows? A. Many times. — Q. Lately. A. It is not a very great while ago that she was here. — Q. Since Christmas; since you came back from

Effra Hall? A. I think I have, or somebody like her. — Q. Have you seen her outside the window? A. Outside the door; not this door, but I mean the gate; we call it a door. — Q. You thought you saw her once or twice outside the house in the Edgware-road? A. No, I have not been able to go there. — Q. But when you were living there, two or three years ago, do you remember living at Mrs. Oldfield's? A. Yes, perfectly well. — Q. Did you see her outside there? A. Yes, I did see her outside there, surrounded by a parcel of policemen: and very lady-like it looked, too. — Q. Anybody else?

(The female who had been requested to leave the room here entered, and said, "Mrs. Cumming was so exhausted last time, that I think she must require a little refreshment."

A Juryman.—What is your name?

Mrs. Cumming (addressing the female who had entered.)—Blake, I want you.

*Dr. Caldwell* (speaking of Mrs. Cumming.)—She wishes to retire; she feels fatigued.

The Commissioner.—We did not think it right to come and trouble you yesterday.

Mrs. Cumming.—I was here ready, and waiting.

The Commissioner.—To-day, Dr. Conolly said you wished us to come. Do not disturb yourself about it; leave yourself in the hands of these gentlemen; you may depend upon it they will do what is right for you.

A Juryman (to Mrs. Cumming.)—The mistake you make, I fear, is, that you think us your enemies; but we are your friends. Every question these gentlemen put to you is for that object; we wish everything should be properly done to you.

Mrs. Cumming.—I am very much obliged to you.

